# Extent of Resection and Long-Term Outcomes for Appendiceal Adenocarcinoma: a SEER Database Analysis of Mucinous and non-Mucinous Histologies

**DOI:** 10.1245/s10434-024-15233-9

**Published:** 2024-04-09

**Authors:** Vasileios Tsagkalidis, Jennie K. Choe, Toni Beninato, Mariam F. Eskander, Miral S. Grandhi, Haejin In, Timothy J. Kennedy, Russell C. Langan, Jason C. Maggi, Henry A. Pitt, H. Richard Alexander, Brett L. Ecker

**Affiliations:** 1 Division of Surgical Oncology, Rutgers Cancer Institute of New Jersey, New Brunswick, NJ USA; 2 Rutgers Robert Wood Johnson Medical School, New Brunswick, NJ USA; 3 Cooperman Barnabas Medical Center, Livingston, NJ USA

**Keywords:** Appendectomy, Appendiceal, Adenocarcinoma, Disease-specific survival hemicolectomy, Lymphadenectomy, Mucinous, Non-mucinous

## Abstract

**Background:**

Mucinous appendiceal adenocarcinomas (MAA) and non-mucinous appendiceal adenocarcinomas (NMAA) demonstrate differences in rates and patterns of recurrence, which may inform the appropriate extent of surgical resection (i.e., appendectomy versus colectomy). The impact of extent of resection on disease-specific survival (DSS) for each histologic subtype was assessed.

**Patients and Methods:**

Patients with resected, non-metastatic MAA and NMAA were identified in the Surveillance, Epidemiology, and End Results database (2000–2020). Multivariable models were created to examine predictors of colectomy for each histologic subtype. DSS was calculated using Kaplan–Meier estimates and examined using Cox proportional hazards modeling.

**Results:**

Among 4674 patients (MAA: *n* = 1990, 42.6%; NMAA: *n* = 2684, 57.4%), the majority (67.8%) underwent colectomy. Among colectomy patients, the rate of nodal positivity increased with higher T-stage (MAA: T1: 4.6%, T2: 4.0%, T3: 17.1%, T4: 21.6%, *p* < 0.001; NMAA: T1: 6.8%, T2: 11.4%, T3: 25.6%, T4: 43.8%, *p* < 0.001) and higher tumor grade (MAA: well differentiated: 7.7%, moderately differentiated: 19.2%, and poorly differentiated: 31.3%; NMAA: well differentiated: 9.0%, moderately differentiated: 20.5%, and 44.4%; *p* < 0.001). Nodal positivity was more frequently observed in NMAA (27.6% versus 16.4%, *p* < 0.001). Utilization of colectomy was associated with improved DSS for NMAA patients with T2 (log rank *p* = 0.095) and T3 (log rank *p* = 0.018) tumors as well as moderately differentiated histology (log rank *p* = 0.006). Utilization of colectomy was not associated with improved DSS for MAA patients, which was confirmed in a multivariable model for T-stage, grade, and use of adjuvant chemotherapy [hazard ratio (HR) 1.00, 95% confidence interval (CI) 0.81–1.22].

**Conclusions:**

Colectomy was associated with improved DSS for patients with NMAA but not MAA. Colectomy for MAA may not be required.

**Supplementary Information:**

The online version contains supplementary material available at 10.1245/s10434-024-15233-9.

Appendiceal adenocarcinoma (AA) is a rare tumor of the gastrointestinal tract.^1,2^ Consensus guidelines for the management of appendiceal adenocarcinoma follows that of colorectal cancer (CRC), given the scarcity of high-quality data and their presumed clinical similarity arising from their common embryologic origin and anatomic proximity.^3–5^ Surgical management of nonmetastatic AA includes appendectomy or right hemicolectomy. Appendectomy alone is currently recommended for patients with intramucosal AA or carcinoma invading the submucosa without invasion of the muscularis propria (T1) and no high-risk features (i.e., low grade, negative resection margins, no lymphovascular invasion). Patients with high-risk T1 tumors or invasion beyond the submucosa (> T2) are considered for a right hemicolectomy for pathologic assessment of the ileocolic lymph nodes.^6,7^

The clinical behavior of appendiceal adenocarcinomas varies on the basis of disease stage and the presence of a mucinous subtype.^8^ Mucinous appendiceal adenocarcinoma (MAA) is distinguished from non-mucinous appendiceal adenocarcinoma (NMAA) by the criterion of having > 50% of the cross-sectional area histologically comprised of mucin.^3^ MAA have distinct patterns of metastatic spread, with mucinous seeding of the peritoneal cavity and a lower rate of lymphatic and hematogenous dissemination.^9,10^ While the rate of isolated lymph node metastases in the absence of other metastatic disease is lower for MAA compared with NMAA, the presence of nodal metastases is nevertheless prognostic of survival for both subtypes.^11,12^ Still, the role of routine colectomy for therapeutic removal of involved lymph nodes may not improve survival, particularly for MAAs. Given the lack of randomized trial data, the appropriate oncologic extent of resection for localized AAs has been guided by several cancer-registry-based analyses, some of which have grouped together both mucinous and non-mucinous histologies, or have been limited by use of overall survival as a study endpoint—where patient comorbidity may bias both extent of resection and the rate of non-disease-related deaths.^13–16^ To clarify the association between extent of resection and disease-specific survival (DSS), a preferrable study endpoint for a cancer with a generally favorably prognosis, the Surveillance, Epidemiology, and End Results (SEER) database was queried to evaluate the oncologic value of the extent of surgical resection for both mucinous and non-mucinous subtypes.

## Patients and Methods

### Patient selection

After institutional review board approval, data (2000–2020) were extracted from the SEER database (https://seer.cancer.gov/). Patients ≥ 18 years of age who underwent resection of appendiceal adenocarcinoma were identified. Patients were selected using appendix site code (ICD-O-3 topography code C18.1) and corresponding ICD-O-3 morphology codes for MAA and NMAA (Supplemental Table 1). Patients were excluded if they had metastatic disease at the time of surgical resection or unknown disease-specific survival.

### Variables

The demographic and clinical SEER variables utilized in this study included age (< 60, 60–69, 70–79, and > 80 years), sex, race/ethnicity, median income, population density (metropolitan areas, adjacent or non-adjacent nonmetropolitan areas), primary tumor (pT)-stage, regional lymph node metastasis (pN)-stage, tumor grade, and administration of adjuvant chemotherapy. Extent of resection was defined by either appendectomy versus hemicolectomy. The primary outcome, DSS, was defined as the interval between date of diagnosis and date of death from disease, with censoring at last contact or death from another cause.

### Statistical Analyses

Descriptive statistics are presented as frequencies for categorical variables and median [interquartile range (IQR)] for continuous variables. Pearson’s *χ*2 and Wilcoxon rank-sum tests were used to analyze categorical and continuous variables, respectively. Variables associated with colectomy on univariate analysis were entered into a stepwise logistic regression model (*p* ≤ 0.05 for entry; *p* > 0.10 for removal) to identify independent predictors of colectomy utilization. The influence of colectomy on DSS was analyzed using Kaplan–Meier estimates and Cox proportional hazards modeling with backwards stepwise selection (*p* ≤ 0.05 for entry; *p* > 0.10 for removal) including all the aforementioned demographic and clinical SEER variables. Multivariable Cox regression was performed both for the overall cohorts and within each T-stage category. Propensity score matching between ‘‘control’’ (i.e., appendectomy) and ‘‘case’’ (i.e., colectomy) was attempted but abandoned owing to significance imbalance between the two groups, highlighting the marked and nonrandom differences in treatment approaches in this national subset.^17,18^ Thus, only multivariable regressions were utilized. *p*-Values ≤ 0.05 were considered statistically significant; all tests were two-sided. Analyses were carried out using SPSS version 29.0 (IBM Corp., Armonk, NY).

## Results

### Rates and Predictors of Colectomy

A total of 7549 patients with appendiceal adenocarcinoma who underwent appendectomy or colectomy were identified in the SEER database. Serial exclusion of patients with metastatic disease at time of initial resection (*n* = 2843) and those without known disease-specific survival (*n* = 32) yielded a final cohort of 4674 patients. The majority (*n* = 3169; 67.8%) underwent colectomy; the remaining patients underwent appendectomy (*n* = 1505; 32.2%). Regarding histologic subtype, 2684 (57.4%) were NMAAs, and 1990 (42.6%) were MAAs.

In both the non-mucinous and mucinous subsets, significant clinicopathologic differences existed between the appendectomy and colectomy-treated groups (Table 1). For both histologies, colectomy was increasingly used for younger patients and was associated with higher T-stages. Notably, no disparities were observed by treatment strategy according to sex, median income, population density, and tumor grade.

**Table 1:** Clinicodemographics of NMAA and MAA patients in the overall study cohort

		Non-mucinous			Mucinous		
		Appendectomy	Colectomy	*p*-Value	Appendectomy	Colectomy	*p*-Value^a^
Age (years)	< 60	264 (31.5%)	789 (42.8%)	< 0.001	290 (43.5%)	627 (47.4%)	< 0.001
	60–69	214 (25.5%)	472 (25.6%)		143 (21.5%)	338 (25.5%)	
	70–79	203 (24.2%)	390 (21.1%)		136 (20.4%)	244 (18.4%)	
	≥ 80	158 (18.8%)	194 (10.5%)		97 (14.6%)	115 (8.7%)	
Sex, female	361 (43%)	852 (46.2%)	0.128	340 (51.1%)	677 (51.1%)	0.972	
Race/ethnicity	NH white	584 (69.6%)	1304 (70.7%)	0.028	466 (70%)	924 (69.8%)	0.483
	NH Black	103 (12.3%)	244 (13.2%)		58 (8.7%)	107 (8.1%)	
	NH Asian/PI	50 (6%)	97 (5.3%)		44 (6.6%)	102 (7.7%)	
	Hispanic^b^	101 (12%)	177 (9.6%)		91 (13.7%)	181 (13.7%)	
	NH American Indian	1 (0.1%)	19 (1%)		2 (0.3%)	7 (0.5%)	
	NH unknown	0 (0%)	4 (0.2%)		5 (0.8%)	3 (0.2%)	
Median income	< $65K	273 (32.5%)	587 (31.8%)	0.746	183 (27.5%)	382 (28.9%)	0.521
	≥ $65K	566 (67.5%)	1257 (68.1%)		483 (72.5%)	942 (71.1%)	
	Unknown	0 (0%)	1 (0.1%)		0 (0%)	0 (0%)	
Population density	Metropolitan	736 (87.7%)	1613 (87.4%)	0.336	593 (89%)	1195 (90.3%)	0.260
	NM, adjacent	65 (7.7%)	132 (7.2%)		38 (5.7%)	80 (6%)	
	NM, nonadjacent	38 (4.5%)	94 (5.1%)		35 (5.3%)	49 (3.7%)	
	Unknown	0 (0%)	6 (0.3%)		0 (0%)	0 (0%)	
T-stage	T1	97 (11.6%)	149 (8.1%)	< 0.001	74 (11.1%)	89 (6.7%)	< 0.001
	T2	120 (14.3%)	220 (11.9%)		67 (10.1%)	102 (7.7%)	
	T3	347 (41.4%)	776 (42.1%)		239 (35.9%)	443 (33.5%)	
	T4	215 (25.6%)	631 (34.2%)		225 (33.8%)	608 (45.9%)	
	TX	60 (7.2%)	69 (3.7%)		61 (9.2%)	82 (6.2%)	
N-stage	N0	633 (75.4%)	1265 (68.6%)	< 0.001	498 (74.8%)	1024 (77.3%)	< 0.001
	N+	101 (12.0%)	496 (26.9%)		60 (9.0%)	204 (15.4%)	
	NX	105 (12.5%)	84 (4.6%)		108 (16.2%)	96 (7.3%)	
Grade^c^	Well	150 (20.4%)	279 (16.8%)	0.095	251 (46.8%)	466 (41.9%)	0.173
	Moderate	407 (55.2%)	942 (56.7%)		224 (41.8%)	507 (45.6%)	
	Poor	180 (24.4%)	441 (26.5%)		61 (11.4%)	138 (12.4%)	
Chemotherapy	Yes	191 (22.8%)	747 (40.5%)	< 0.001	162 (24.3%)	521 (39.4%)	< 0.001
	No/unknown	648 (77.2%)	1098 (59.5%)		504 (75.7%)	803 (60.6%)	

*NH* non-Hispanic, *PI* Pacific Islander, *NM* nonmetropolitan

^a^Pearson’s Chi-squared test

^b^All Hispanic races included

^c^Grade not available for 265 (10.6%) and 324 (17.0%) of non-mucinous and mucinous cases, respectively

Data on the number of lymph nodes examined were available in 4579 patients (98% of study cohort). Patients who underwent appendectomy had fewer lymph nodes examined (median 1, IQR 0–13) compared with colectomy patients (median 16, IQR 11–22; *p* < 0.001), including the subset of patients with N-positive (N+) disease (*p* < 0.001). In total, 161 patients underwent appendectomy alone for N-positive disease, and compared with colectomy patients (of any nodal staging) they were not significantly different with regards to age (*p* = 0.506), race/ethnicity (*p* = 0.555), median income (*p* = 0.974), or population density (*p* = 0.774). However, they were more likely to be male (61.5% versus 51.8%, *p* = 0.016) and more likely to receive adjuvant chemotherapy (64.6% versus 40.0%, *p* < 0.001).

Preoperative carcinoembryonic antigen (CEA) values were available for 25.6% of the overall cohort (non-mucinous: *n* = 670; mucinous: *n* = 525); in both subsets, elevated CEA was not associated with rates of colectomy (*p* = 0.400 and *p* = 0.605, respectively). In a multivariable logistic regression model, age, T-stage, and N-stage were independently associated with colectomy use (Supplemental Table 2).

### Predictors of Lymph Node Positivity

Given that T-stage was associated with extent of surgery, the risk of lymph node metastases was quantified for increasing T-categories among colectomy patients. For non-mucinous histologies, the rate of nodal positivity increased with increasing T-stage (T1: 6.8%, T2: 11.4%, T3: 25.6%, and T4: 43.8%, *p* < 0.001). Likewise, for mucinous histologies, the rate of nodal positivity increased with increasing T-stage (T1: 4.6%, T2: 4.0%, T3: 17.1%, and T4: 21.6%, *p* < 0.001). Overall, the rate of nodal positivity was greater for non-mucinous histology (27.6% versus 16.4%, *p* < 0.001).

Since tumor grade has been previously reported to be associated with risk of lymph node disease, rates of lymph node metastases in NMAA and MAA were analyzed in our cohort, stratified by tumor grade.^11,19^ For both histologies, grade was significantly associated with N+ disease (*p* < 0.001), with poorly differentiated tumors exhibiting the highest rates of lymph node metastasis (NMAA: 44.4%, MAA: 31.3%, *p* = 0.002; Supplemental Table 3). Patients with well-differentiated and moderately differentiated tumors had lower rates of lymph node metastases, with no significant difference between NMAA and MAA tumors (well differentiated: 9.0% versus 7.7%, *p* = 0.450; poorly differentiated: 20.5% versus 19.2%, *p* = 0.490, respectively).

### Influence of Colectomy on Disease-Specific Survival in the Overall Cohort

Median follow-up was 74 (IQR 33–131) months and 87 (IQR 41–150) months for the non-mucinous and mucinous adenocarcinoma subsets, respectively. A total of 1214 (26.0%) disease-specific deaths were observed. The long-term survival impact of extent of surgical resection was examined using Cox proportional hazards modeling, including those variables significantly associated with DSS by univariate analysis (Table 2). In both histologic subsets, variables independently associated with DSS included age, T-stage, N-stage, and grade. Utilization of colectomy was not associated with DSS in the non-mucinous [ hazard ratio (HR) 0.88, 95% confidence interval (CI) 0.75–1.03; adjusted *p*-value 0.107] nor mucinous (HR 0.95, 95% CI 0.78–1.17; adjusted *p*-value 0.963) subsets. Regardless of extent of surgical resection, prognosis was excellent for both non-mucinous [appendectomy: mean 164.1 (95% CI 155.4–172.9) months; colectomy: 168.4 (95% CI 162.6–174.3) months; log rank *p* = 0.209] and mucinous [mean 181.6 (95% CI 172.4–190.8) months; 181.7 (95% CI 175.3–188.1) months, log rank *p* = 0.801] adenocarcinoma patients.

**Table 2:** Cox regression model for independent predictors of disease-specific survival

		Non-mucinous				Mucinous			
		Univariate		Multivariate		Univariate		Multivariate	
		HR	95% CI	HR	95% CI	HR	95% CI	HR	95% CI
Age (years)	< 60	Ref	Ref	Ref	Ref	Ref	Ref	Ref	Ref
	60–69	1.19	0.99–1.44	1.14	0.94–1.38	1.18	0.93–1.49	1.28	1.01–1.62
	70–79	1.38	1.14–1.67	1.54	1.26–1.86	1.70	1.35–2.16	1.72	1.36–2.18
	≥ 80	2.14	1.73–2.66	2.65	2.13–3.31	2.53	1.90–3.37	2.68	2.01–1.60
Sex, female		1.01	0.88–1.17	–	–	0.78	0.65–0.94	0.75	0.62–0.90
Race/ethnicity	NH white	Ref	Ref	Ref	Ref	Ref	Ref	Ref	Ref
	Other	1.14	0.98–1.34	1.25	1.07–1.47	0.95	0.78–1.17	–	–-
Median income	< $65K	Ref	Ref	Ref	Ref	Ref	Ref	Ref	Ref
	≥ $65K	0.95	0.81–1.10	–	–	0.84	0.69–1.01	–	–
Population density	Metropolitan	Ref	Ref	Ref	Ref	Ref	Ref	Ref	Ref
	NM, adjacent	1.01	0.77–1.34	–	–	1.16	0.80–1.69	–	–
	NM, nonadjacent	0.82	0.57–1.16	–	–	1.30	0.86–1.96	–	–
T-stage	T1	Ref	Ref	Ref	Ref	Ref	Ref	Ref	Ref
	T2	1.65	1.04–2.61	1.59	1.01–2.52	0.85	0.40–1.69	0.80	0.40–1.59
	T3	2.39	1.60–3.57	2.00	1.33–3.01	2.31	1.42–3.77	1.87	1.14–3.06
	T4	5.49	3.68–8.17	3.99	2.65–6.01	3.82	2.37–6.17	3.14	1.93–5.09
N-stage	N0	Ref	Ref	Ref	Ref	Ref	Ref	Ref	Ref
	N+	3.08	2.63–3.60	2.29	1.94–2.71	2.78	2.23–3.47	2.05	1.63–2.59
Grade	Well	Ref	Ref	Ref	Ref	Ref	Ref	Ref	Ref
	Moderate	1.40	1.07–1.75	1.17	0.92–1.50	1.86	1.49–2.33	1.63	1.30–2.05
	Poor	2.63	2.04–3.39	1.70	1.30–2.21	2.45	1.82–3.30	1.94	1.43–2.63
Chemotherapy	No	Ref	Ref	Ref	Ref	Ref	Ref	Ref	Ref
	Yes	1.66	1.43–1.92	–	–	1.52	1.27–1.82	–	–
Surgical extent	Appendectomy	Ref	Ref	Ref	Ref	Ref	Ref	Ref	Ref
	Colectomy	0.91	0.78–1.06	0.88	0.75–1.03	1.03	0.84–1.25	0.95	0.78–1.17

*NH* non-Hispanic, *NM* nonmetropolitan

### Survival Analysis of Extent of Resection, Stratified by T-stage

**Fig. 1:**
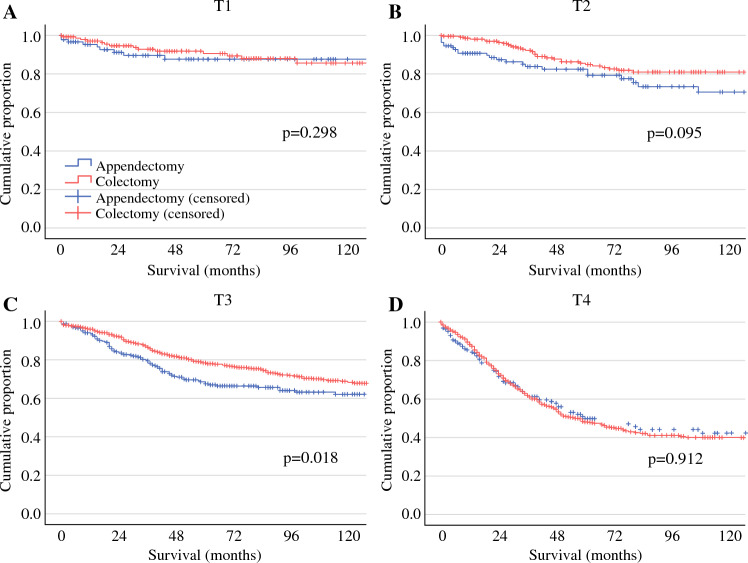
Impact of appendectomy versus colectomy on disease-specific survival in patients with NMAA, stratified by T-stage*. NMAA* non-mucinous appendiceal adenocarcinoma

Given that T-stage and N-stage were each associated with disease-specific survival, and that nodal positivity was increasingly observed for higher T-stage, the impact of extent of surgery was analyzed for each T-stage. For non-mucinous appendiceal adenocarcinoma, there was no difference in DSS observed for appendectomy versus colectomy for patients with T1 tumors (log rank *p* = 0.298; Fig. 1). In contrast, utilization of colectomy was associated with improved DSS for patients with T3 (log rank *p* = 0.018) tumors and a trend toward improved DSS was observed in patients with T2 tumors (log rank *p* = 0.095). Patients with T4 non-mucinous tumors managed with appendectomy or colectomy evidenced similar DSS (log rank *p* = 0.912). In a multivariable cox regression accounting for age, N-stage, grade, and use of adjuvant chemotherapy, colectomy in patients with T3 tumors was associated with reduced risk of disease-specific death (HR 0.75, 95% CI 0.58–0.98, *p* = 0.032). Extent of surgery was not independently associated with DSS for other T categories (*p* = 0.495, *p* = 0.100, and *p* = 0.259 for T1, T2, and T4 lesions, respectively).

**Fig. 2:**
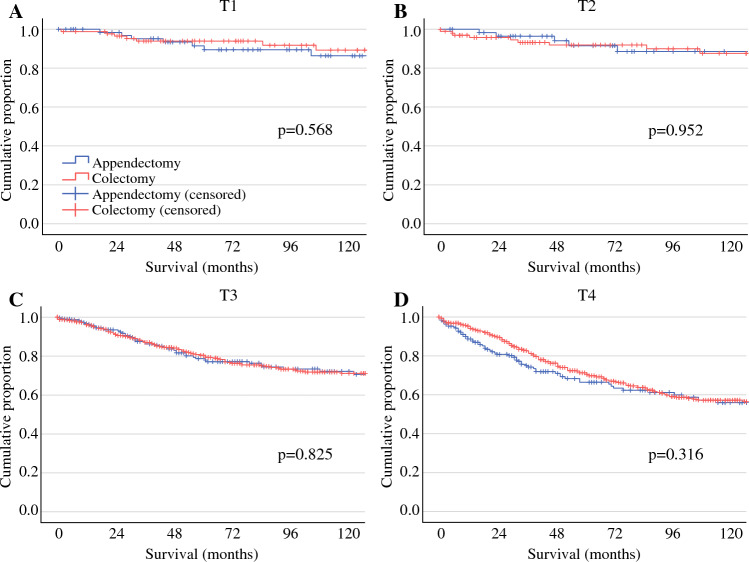
Impact of appendectomy versus colectomy on disease-specific survival in patients with MAA, stratified by T-stage*. MAA* mucinous appendiceal adenocarcinoma

In contrast, for patients with mucinous tumors (Fig. 2), no difference in DSS was observed for appendectomy versus colectomy for any of the T-categories (T1: *p* = 0.568; T2: *p* = 0.952; T3: *p* = 0.825; and T4: *p* = 0.316). Multivariable Cox regression, accounting for age, N-stage, grade, and use of adjuvant chemotherapy, also demonstrated no difference between appendectomy and colectomy-treated groups (*p* = 0.169, *p* = 0.620, *p* = 0.761, and *p* = 0.310 for T1, T2, T3, and T4 lesions, respectively).

### Survival Analysis of Extent of Resection, Stratified by Grade

**Fig. 3:**
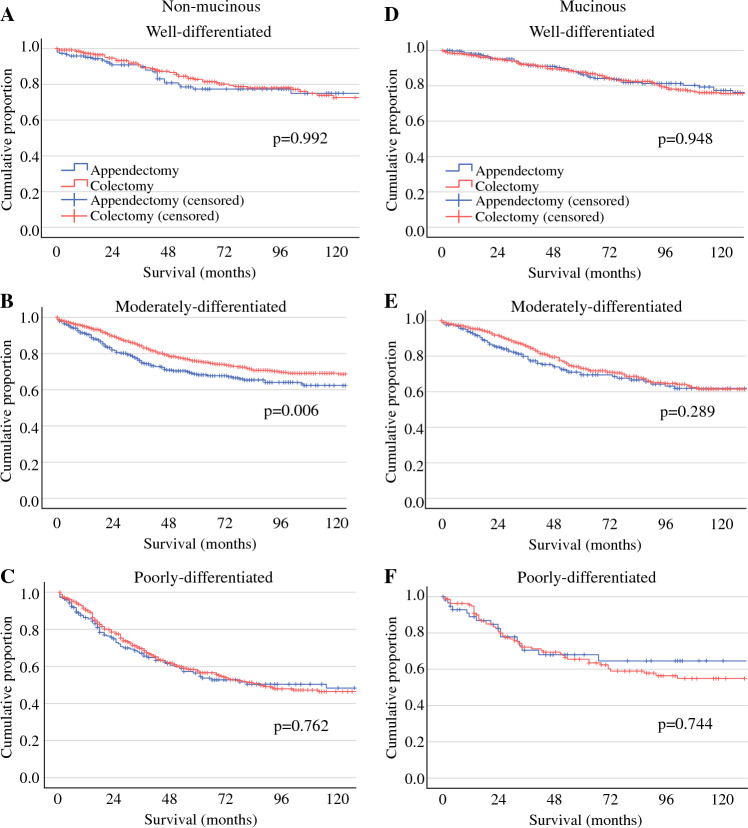
Impact of appendectomy versus colectomy on disease-specific survival in patients with NMAA and MAA, stratified by grade*. NMAA* non-mucinous appendiceal adenocarcinoma, *MAA* mucinous appendiceal adenocarcinoma

The association of the extent of surgical resection and DSS was subsequently analyzed for each tumor type and stratified by tumor grade. For patients with moderately differentiated NMAA, hemicolectomy was associated with improved DSS compared with appendectomy (log rank *p* = 0.006; Fig. 3). In contrast, colectomy was not associated with DSS in well-differentiated (log rank *p* = 0.992) and poorly differentiated (log rank *p* = 0.762) NMAA. For patients with MAA, extent of resection was not associated with DSS for any tumor grade (well differentiated: *p* = 0.948, moderately differentiated: *p* = 0.289, poorly differentiated: *p* = 0.744).

## Discussion

The current study comprises the largest population-based study evaluating disease-specific survival by extent of resection for patients with mucinous and non-mucinous subtypes of appendiceal adenocarcinoma. In our analysis, non-mucinous histologies were associated with significant rates of lymph node metastases (26–44% for T3–4 lesions). In support of expert guidelines, surgical clearance of such lymph nodes with colectomy was associated with improvements in cancer-related deaths, particularly for T3 and moderately differentiated tumors. In contrast, mucinous histologies had lower rates of lymph node metastases, and use of colectomy was not associated with improved disease-specific survival for any MAA patients. Such data confirm the divergent biologic characteristics of mucinous versus non-mucinous AA and highlight the importance of consideration of histologic subtype in their surgical management.

Previous SEER-based propensity-score-matched analysis found that mucinous histology was not independently associated with long-term survival in stage I–III AA patients.^20^ The authors subsequently conclude that the same treatment strategies can be applied regardless of histologic subtype. However, similar risk-adjusted prognosis does not necessarily equate to similar surgical treatment. Here, by segregating the NMAA and MAA patients in all statistical analyses, a histology-dependent association between extent of resection and disease-specific survival was identified. For patients with NMAA, absolute improvements in DSS were observed for T2 and T3 lesions as well as moderately differentiated tumors. A DSS advantage was not observed in patients with T1 tumors. In support, previous studies have not demonstrated a survival benefit of an extensive lymphadenectomy for low risk T1 tumors, likely given the low rates of nodal disease.^14,15^ Patients with T1 tumors exhibited lymph node positivity rates of only 6.8% when undergoing colectomy. The lack of association between extent of surgery and survival for NMAA T4 tumors was an interesting finding, possibly driven by the higher risk of metastatic failure that would render extensive lymphadenectomy alone an ineffective way to achieve cure.

Oncologic tenets from the surgical management of colorectal cancer—namely, the use of colectomy for surgical staging of the draining lymph node basin—may not be relevant for the mucinous subtype of AA. In support, genomic analyses have revealed differences in the mutational landscape of AA and CRC as well as between MAA and NMAA.^21^ Compared with NMAA, MAA are more likely to harbor mutations in *KRAS* and *GNAS*, with fewer mutations in *TP53*.^22,23^ These genomic differences provide biologic support for consideration of distinct clinical entities requiring individualized treatment approaches. In these data, disease-specific survival was independent of type of resection for all MAA patients in a multivariable model adjusting for T-stage, grade, and use of adjuvant chemotherapy. Likewise, a previous analysis of MAA using the SEER dataset found that extent of resection was not associated with disease-specific survival, although this prior analysis involved a more heterogeneous cohort inclusive of patients with metastatic disease and data dating primarily from the twentieth century.^24^ Still, the current results were surprising, given the significant risk of nodal involvement for certain patients (17–22% in T3 and T4 MAA; 18–29% for moderately and poorly differentiated MAA).

One possible explanation could be the unique biologic behavior of mucinous tumors. These tumors have a propensity to recur within the peritoneum, which may not be prevented by more extensive lymphadenectomy. Mucin 2 (MUC2) is the most abundant, gel-forming, mucus protein primarily secreted in the small bowel and colon.^25^ MUC2 and MUC5A have been found to be overexpressed in MAA, with MUC2 being more profoundly expressed in pseudomyxoma peritonei of appendiceal origin.^26,27^ Overexpression of MUC2 has been associated with decreased survival in multiple cancer types.^28–31^ Under normal conditions, secreted mucin serves to protect the intestinal epithelium and subsequently undergoes degradation. However, when peritoneal seeding occurs, the produced mucin fails to degrade within the peritoneal cavity, leading to accumulation and development of pseudomyxoma peritonei. This mucin can shield cancer cells from the host’s immune system as well as prevent the delivery of chemotherapy agents, leading to treatment failure.^32,33^ Additionally, the mucin can facilitate the spread of tumor cells within the peritoneal cavity and create a favorable microenvironment that enhances tumor growth.^33,34^ This unique biologic behavior potentially renders an extensive lymphadenectomy alone an ineffective way to achieve cure for localized MAA. Relatedly, in a study examining outcomes in patients with metastatic MAA and peritoneal seeding who underwent cytoreductive surgery and intraperitoneal chemotherapy, the addition of right hemicolectomy did not provide a survival advantage over those who underwent appendectomy alone. Additionally, when the cohort was stratified by lymph node status, no difference in survival was observed between node-negative patients, node-positive patients, and patients with unknown nodal status.^35^

Without the prospect of randomized data given the rarity of this entity, these multivariable-adjusted registry-based data may be the best quality data available to guide clinical management. Still, these findings need to be interpreted with caution, and several limitations warrant emphasis. Cox regression models were used to adjust for known patient-related (i.e., race/ethnicity) and tumor-related confounders.^8,22,36–39^ Nevertheless, significant differences were observed between the appendectomy and the colectomy cohorts. An attempt to build propensity-score-matched cohorts failed, which points to the likely impact of treatment bias present in these nonrandomized data. Still, SEER reporting of disease-specific (versus overall) survival allowed for more precise understanding of the impact of surgical extent on long-term outcomes while minimizing the bias of patient comorbidity on both surgical treatment and the rate of non-disease-related deaths. A second major limitation is the lack of data on resection margin status following appendectomy as well as information on specific histologic characteristics, such as lymphovascular invasion (LVI). These two variables are important determinants in surgical management of T1 tumors; without these data, our findings may only be applicable for T1 tumors without positive resection margins or LVI. Third, the analyses of tumor grade are limited by institutional differences in pathologic grading as well as changes in the grade classification (i.e., two- versus three- versus four-tier system) that occurred over the study period. Thus, the results of the grade-stratified analyses need to be validated in additional contemporary series. Fourth, serum biomarkers (e.g., Ca 19-9 and CA 125) may be elevated in patients with appendiceal adenocarcinoma and have been associated with survival. Unfortunately, such data were not available to be included in these analyses. Lastly, limitations particular to SEER include certain database inadequacies, such as (a) specific chemotherapy regimens utilized, (b) completion of prescribed treatment schedules, (c) rates and types of recurrences, and (d) all clinical details that may have informed decisions regarding extent of surgery. In this regard, we observed a small number of patients who underwent appendectomy alone for node-positive disease that could not be explained by differences in their social determinants of health. It is possible that patient comorbidity may have contributed to some of these treatment decisions.

## Conclusions

In this contemporary, population-based analysis of the use of colectomy for nonmetastatic mucinous and non-mucinous appendiceal adenocarcinoma, the survival impact of surgical strategies varied by histologic subtype. No improvement in disease-free survival was observed for the subset of patients with mucinous AA regardless of T-stage and histologic grade. These data support consideration of histologic subtype in the surgical treatment of localized AA. In the absence of randomized data, these results raise doubts regarding the need for colectomy for localized MAA.

### Supplementary Information

Below is the link to the electronic supplementary material.

Supplementary file1 (DOCX 12 KB)

Supplementary file2 (DOCX 14 KB)

Supplementary file3 (DOCX 15 KB)

## References

[1] Marmor S, Portschy PR, Tuttle TM, Virnig BA (2015). The rise in appendiceal cancer incidence: 2000–2009. J Gastrointest Surg..

[2] Singh H, Koomson AS, Decker KM, Park J, Demers AA (2020). Continued increasing incidence of malignant appendiceal tumors in Canada and the United States: a population-based study. Cancer..

[3] Raghav K, Shen JP, Jacome AA (2020). Integrated clinico-molecular profiling of appendiceal adenocarcinoma reveals a unique grade-driven entity distinct from colorectal cancer. Br J Cancer..

[4] Chicago Consensus Working G (2020). The Chicago consensus on peritoneal surface malignancies: management of appendiceal neoplasms. Ann Surg Oncol..

[5] Glasgow SC, Gaertner W, Stewart D (2019). The American society of colon and rectal surgeons, clinical practice guidelines for the management of appendiceal neoplasms. Dis Colon Rectum..

[6] Van de Moortele M, De Hertogh G, Sagaert X, Van Cutsem E (2020). Appendiceal cancer: a review of the literature. Acta Gastroenterol Belg. Jul-Sep.

[7] National Comprehensive Cancer Network—Colon Cancer—Version 2.2023. Accessed 9/18/2023. https://www.nccn.org/professionals/physician_gls/pdf/colon.pdf

[8] Nitecki SS, Wolff BG, Schlinkert R, Sarr MG (1994). The natural history of surgically treated primary adenocarcinoma of the appendix. Ann Surg..

[9] Kabbani W, Houlihan PS, Luthra R, Hamilton SR, Rashid A (2002). Mucinous and nonmucinous appendiceal adenocarcinomas: different clinicopathological features but similar genetic alterations. Mod Pathol..

[10] Minhas A, Hendrickson J, Minhas SA (2021). Frequency and risk factors for metastasis in newly diagnosed appendiceal carcinoma. Cureus..

[11] Shannon AB, Goldberg D, Song Y (2020). Predictors of lymph node metastases in patients with mucinous appendiceal adenocarcinoma. J Surg Oncol..

[12] Davison JM, Choudry HA, Pingpank JF (2014). Clinicopathologic and molecular analysis of disseminated appendiceal mucinous neoplasms: identification of factors predicting survival and proposed criteria for a three-tiered assessment of tumor grade. Mod Pathol..

[13] Elias H, Galata C, Warschkow R (2017). Survival after resection of appendiceal carcinoma by hemicolectomy and less radical than hemicolectomy: a population-based propensity score matched analysis. Colorectal Dis..

[14] AlMasri SS, Hammad AY, Singhi AD (2023). Appendectomy is oncologically equivalent to right hemicolectomy for well-differentiated T1 appendiceal adenocarcinoma. Dis Colon Rectum..

[15] Straker RJ, Grinberg SZ, Sharon CE (2022). Pathologic factors associated with low risk of lymph node metastasis in nonmucinous adenocarcinoma of the appendix. Ann Surg Oncol..

[16] Marks VA, Kerekes D, Butensky S (2023). Role of colectomy in the management of appendiceal tumors: a retrospective cohort study. BMC Gastroenterol..

[17] Austin PC (2014). The use of propensity score methods with survival or time-to-event outcomes: reporting measures of effect similar to those used in randomized experiments. Stat Med..

[18] Austin PC (2011). An introduction to propensity score methods for reducing the effects of confounding in observational studies. Multivar Behav Res..

[19] Turner KM, Morris MC, Delman AM (2022). Do lymph node metastases matter in appendiceal cancer with peritoneal carcinomatosis? A US HIPEC collaborative study. J Gastrointest Surg..

[20] Widmann B, Warschkow R, Schmied BM, Marti L, Steffen T (2016). Impact of mucinous histology on the prognosis of stage I-III adenocarcinomas of the appendix: a population-based, propensity score-matched analysis. J Gastrointest Surg..

[21] Tokunaga R, Xiu J, Johnston C (2019). Molecular profiling of appendiceal adenocarcinoma and comparison with right-sided and left-sided colorectal cancer. Clin Cancer Res..

[22] Ang CS, Shen JP, Hardy-Abeloos CJ (2018). Genomic landscape of appendiceal neoplasms. JCO Precis Oncol..

[23] Foote MB, Walch H, Chatila W (2023). Molecular classification of appendiceal adenocarcinoma. J Clin Oncol..

[24] Turaga KK, Pappas S, Gamblin TC (2013). Right hemicolectomy for mucinous adenocarcinoma of the appendix: just right or too much?. Ann Surg Oncol..

[25] Amini A, Masoumi-Moghaddam S, Ehteda A, Morris DL (2014). Secreted mucins in pseudomyxoma peritonei: pathophysiological significance and potential therapeutic prospects. Orphanet J Rare Dis..

[26] O'Connell JT, Hacker CM, Barsky SH (2002). MUC2 is a molecular marker for pseudomyxoma peritonei. Mod Pathol..

[27] Shibahara H, Higashi M, Yokoyama S (2014). A comprehensive expression analysis of mucins in appendiceal carcinoma in a multicenter study: MUC3 is a novel prognostic factor. PLoS ONE..

[28] He YF, Zhang MY, Wu X (2013). High MUC2 expression in ovarian cancer is inversely associated with the M1/M2 ratio of tumor-associated macrophages and patient survival time. PLoS ONE..

[29] Kasashima S, Kawashima A, Zen Y (2007). Expression of aberrant mucins in lobular carcinoma with histiocytoid feature of the breast. Virchows Arch..

[30] Perez RO, Bresciani BH, Bresciani C (2008). Mucinous colorectal adenocarcinoma: influence of mucin expression (Muc1, 2 and 5) on clinico-pathological features and prognosis. Int J Colorectal Dis..

[31] Hugen N, Simons M, Halilovic A (2015). The molecular background of mucinous carcinoma beyond MUC2. J Pathol Clin Res..

[32] Dilly AK, Honick BD, Lee YJ (2017). Targeting G-protein coupled receptor-related signaling pathway in a murine xenograft model of appendiceal pseudomyxoma peritonei. Oncotarget..

[33] Hollingsworth MA, Swanson BJ (2004). Mucins in cancer: protection and control of the cell surface. Nat Rev Cancer..

[34] Kufe DW (2009). Mucins in cancer: function, prognosis and therapy. Nat Rev Cancer..

[35] Gonzalez-Moreno S, Sugarbaker PH (2004). Right hemicolectomy does not confer a survival advantage in patients with mucinous carcinoma of the appendix and peritoneal seeding. Br J Surg..

[36] Grotz TE, Royal RE, Mansfield PF (2017). Stratification of outcomes for mucinous appendiceal adenocarcinoma with peritoneal metastasis by histological grade. World J Gastrointest Oncol..

[37] Asare EA, Compton CC, Hanna NN (2016). The impact of stage, grade, and mucinous histology on the efficacy of systemic chemotherapy in adenocarcinomas of the appendix: analysis of the National Cancer Database. Cancer..

[38] Miller KD, Nogueira L, Devasia T (2022). Cancer treatment and survivorship statistics, 2022. CA Cancer J Clin..

[39] Zavala VA, Bracci PM, Carethers JM (2021). Cancer health disparities in racial/ethnic minorities in the United States. Br J Cancer..

