# Supplementary material for: Extent of Resection and Long-Term Outcomes for Appendiceal Adenocarcinoma: a SEER Database Analysis of Mucinous and non-Mucinous Histologies
Source: Ann Surg Oncol. 2024 Apr 9;31(7):4203–12. doi: 10.1245/s10434-024-15233-9 (PMC11164803; doi:10.1245/s10434-024-15233-9)
Supplement: Supplementary file 1 — Supplementary file1 (DOCX 12 KB) [file 10434_2024_15233_MOESM1_ESM.docx]

| **Supplemental Table 1**. Histologic type ICD-O-3 | |
| --- | --- |
| Non-mucinous | 8140; 8144; 8210; 8211; 8220; 8255; 8260; 8261; 8262; 8263 8323; 8440; 8460; 8490; 8560; 8574 |
| Mucinous | 8470; 8472; 8480; 8481 |
