# Supplementary material for: Extent of Resection and Long-Term Outcomes for Appendiceal Adenocarcinoma: a SEER Database Analysis of Mucinous and non-Mucinous Histologies
Source: Ann Surg Oncol. 2024 Apr 9;31(7):4203–12. doi: 10.1245/s10434-024-15233-9 (PMC11164803; doi:10.1245/s10434-024-15233-9)
Supplement: Supplementary file 2 — Supplementary file2 (DOCX 14 KB) [file 10434_2024_15233_MOESM2_ESM.docx]

| **Supplemental Table 2.** Binary Logistic Regression Model for Utilization of Colectomy in Management of Appendiceal Adenocarcinoma | | | | | | | |
| --- | --- | --- | --- | --- | --- | --- | --- |
|  | | **Non-mucinous** | | | **Mucinous** | | |
|  |  | **OR** | **95% CI** | **p-value** | **OR** | **95% CI** | **p-value** |
| Age | <60 | Ref | Ref | <0.001 | Ref | Ref | <0.001 |
|  | 60-69 | 0.72 | 0.58-0.90 | 0.003 | 1.07 | 0.84-1.37 | 0.568 |
|  | 70-79 | 0.65 | 0.52-0.81 | <0.001 | 0.82 | 0.63-1.05 | 0.118 |
|  | ≥80 | 0.41 | 0.32-0.54 | <0.001 | 0.55 | 0.41-0.75 | <0.001 |
| T-stage | T1-T2s | Ref | Ref | --- | Ref | Ref | --- |
|  | T3-T4 | 1.21 | 0.99-1.49 | 0.063 | 1.63 | 1.27-2.09 | <0.001 |
| N-stage | N0 | Ref | Ref | --- | Ref | Ref | --- |
|  | N+ | 2.32 | 1.83-2.96 | <0.001 | 1.52 | 1.11-2.07 | 0.009 |
