# Supplementary material for: Extent of Resection and Long-Term Outcomes for Appendiceal Adenocarcinoma: a SEER Database Analysis of Mucinous and non-Mucinous Histologies
Source: Ann Surg Oncol. 2024 Apr 9;31(7):4203–12. doi: 10.1245/s10434-024-15233-9 (PMC11164803; doi:10.1245/s10434-024-15233-9)
Supplement: Supplementary file 3 — Supplementary file3 (DOCX 15 KB) [file 10434_2024_15233_MOESM3_ESM.docx]

| **Supplemental Table 3**. Lymph node status of NMAA and MAA, stratified by tumor grade.^a^ | | | | | | | |
| --- | --- | --- | --- | --- | --- | --- | --- |
|  | | **Non-mucinous** | | | **Mucinous** | | |
|  |  | **N0** | **N+** | **p-value^b^** | **N0** | **N+** | **p-value^b^** |
| Grade | G1 | 364 (91.0%) | 36  (9.0%) | <0.001 | 589 (92.3%) | 49  (7.7%) | <0.001 |
|  | G2 | 1011 (79.5%) | 261  (20.5%) |  | 551 (80.8%) | 131  (19.2%) |  |
|  | G3 | 320 (55.6%) | 256  (44.4%) |  | 125 (68.7%) | 57  (31.3%) |  |
| ^a^ Excluded patients with unknown nodal status (NMAA n=151, MAA n=145)  ^b^ Pearson’s Chi-squared test | | | | | | | |
